# Proceedings Northern Ohio Dental Society

**Published:** 1884-07

**Authors:** W. P. Horton


### Proceedings of Northern Ohio Dental Society.

REPORTED FOR THE DENTAL REGISTER.

BY DR. W. P. HORTON.

The twenty-fifth annual meeting of the Northern Ohio Dental Association began in the assembly room of the Board of Education, on Euclid Avenue, Cleveland, May 13, 1884, at 10:30 o'clock A. M., and continued two days. There was a good attendence of members, both active and honorary, as well as visitors of the profession from other States.

The President, Dr. Gale French, of Pittsburgh, Pa., called the meeting to order promptly at the hour. Most of the morning session was taken up in calling the roll, hearing reports of the several standing committees, and other miscellaneous business.

The time fixed for holding the sessions after the first morning was from 9 A. M. to 12 M.; and from 2 to 5, and 7:30 P. M. to adjournment.

Letters of regret were read from Dr. Thomas, Detroit, Mich., and Dr. E. J. Waye, of Sandusky. The President then announced the first subject on the programme as in order for discussion, viz., "The Causes of Failures in Dental Operations." But the time for adjournment being so near, consideration of the subject was postponed until after recess. On assembling in the afternoon, Drs. J. R. Bell and W. P. Horton, of Cleveland, and Dr. B. F. Gibbons, of Youngstown, read papers upon the first subject, after which a very lively and interesting discussion ensued, participated in by Drs. J. W. Lyder, C. R. Butler, Geo. H. Wilson, J. F. Liddall, D. Gibbons, F. S. Whitslar, W. P. Horton, B. F. Gibbons, Corydon Palmer, V. McAlpine, J. R. Bell, Dr. Elliott, of Meadeville, Pa., and L. Buffet, which occupied the time to the hour for adjournment.

At the opening of the evening session, the first subject was passed, and the second taken up, viz.: "Materials for Filling Teeth, and How to Use Them." Upon this topic there were no papers; but the remarks of members and visitors were spirited and instructive to the close of the session.

May 14, 9 A. M., the meeting was called to order by the President, Dr. French. Drs. P. H. Keese and S. B. Dewey, both of Cleveland, were elected active members. During the consideration of miscellaneous business, Dr. J. F. Liddall read an original poem on anæsthetics, which was well received. The discussion of the second subject was continued to 10 o'clock A. M., the time fixed for the election of officers, which resulted in the choice for President, of Dr. Ira Brown, of Cleveland ; Vice-President, Dr. D. Gibbons, of Warren ; Corresponding Secretary, Dr. F. H. Harvey, of Cleveland ; Recording Secretary, Dr. Geo. H. Wilson, of Painesville ; Treasurer, Dr. Charles Buffet, Cleveland.

Delegates to the American Dental Association, which meets at Saratoga Springs the fifth day in August, are: Drs. Gale French, Ira Brown, Phillip A. Keese, Geo. H. Wilson, E. J. Waye, J. W. Lyder, F. S. Whitslar, and C. R. Butler.

There was a manifest desire on the part of members that the society should be fully represented at the next meeting of the American Dental Society, and Dr. Bell was appointed a committee of one to confer with railroad officials, if possible to obtain reduced rates of fare for all who desire to attend. Here the second subject was passed and the third, viz., "Prosthetic Dentistry, including Artificial Crowns" was announced as before the meeting for discussion. Although an old and hackneyed subject, many good, and some new and valuable suggestions were made upon it. At 2 o'clock P. M. the gavel of President French called the meeting to order, and the President-elect, Dr. Ira Brown, was duly installed by a formal introduction by the chairman of a special committee appointed for that purpose. Dr. Brown thanked the members for the honor they had conferred on him in a very brief, but neatly worded speech, at the close of which Dr. Gale French, the retiring President, delivered a very carefully prepared address, giving the history chronologically of this society from its organization to the present time, which was received with hearty applause. The third subject being under consideration, after remarks by several members Dr. Corydon Palmer exhibited some splendidly prepared colored drawings showing his style and method of constructing and antagonizing partial and complete sets of artificial teeth. This embraced both the practical and esthetical, showing prosthetic dentistry to be a fine art. These drawings ought to be obtained by some enterprising book maker, and published for the benefit of the profession.

There were interesting and instructive remarks made by several members on the benefits to be derived from using the Coffin split plate and fixture for reducing irregularities. The third subject was here passed, and the fourth subject, viz., "Subjects Proposed by Members and Quiz," announced as before the meeting.

At this point the special committee on the demise of Dr. B. F. Robinson asked and obtained leave to report as follows, viz:

WHEREAS, Dr. B. F. Robinson, one of the charter members of the Northern Ohio Dental Association, having earnestly and faithfully fulfilled his mission, and passed away to that bourne from which no traveler returns, therefore

*Resolved*, That in his decease the Association has sustained the loss of a valuable and esteemed member, one whose memory will be cherished with feelings of the kindest respect; and that this expression of our regard be placed on record, and a copy sent to the widow of the deceased.

F. S. WHITSLAR,<br>L. BUFFET,<br>J. W. LYDER.

} *Committee.*

This was adopted. The President announced the Executive Committee to be Drs. Whitslar and Whiteside, of Youngstown, and Dr. Barnes, of Cleveland. Dr. Arthur extended a cordial invitation to the members to attend the next meeting of the Odontological Society of Pennsylvania.

The time having arrived for bringing the session to a close, on motion adjourned to meet at Youngstown on the second Tuesday of May, 1885, all present expressing themselves as more than pleased with the good and profitable time they had enjoyed.

